# Supplementary material for: Investigating the long-term impact of misinformation interventions in upper secondary education
Source: PLoS One. 2025 Jul 9;20(7):e0326928. doi: 10.1371/journal.pone.0326928 (PMC12240385; doi:10.1371/journal.pone.0326928)
Supplement: S1 File — (DOCX) [file pone.0326928.s001.docx]

**S1: Supporting information**

Pre-registration: https://osf.io/wzjgm/?view_only=a1f19225cafc4636a5f2aaa2adb24cef

Teaching materials: https://osf.io/fnp8u/files/osfstorage

Data and R code are available at: https://osf.io/fnp8u/files/osfstorage
